# Treatment of Tinnitus in Children—A Systematic Review

**DOI:** 10.3389/fneur.2021.726803

**Published:** 2021-09-10

**Authors:** Max J. Dullaart, Marijn Kip, Adriana L. Smit, Inge Stegeman

**Affiliations:** ^1^Department of Otorhinolaryngology, Head and Neck Surgery, University Medical Center Utrecht, Utrecht, Netherlands; ^2^University Medical Center Utrecht Brain Center, University Medical Center Utrecht, Utrecht, Netherlands; ^3^Epidemiology and Data Science, Amsterdam University Medical Centers, University of Amsterdam, Amsterdam, Netherlands

**Keywords:** tinnitus, subjective, children, treatment, review, systematic

## Abstract

**Objectives:** To systematically review studies on the effect of treatment of subjective tinnitus in children.

**Data Sources:** We searched for studies in MEDLINE and EMBASE databases, after which additional studies were hand searched using Scopus databases. The methods are described in the study protocol, which has been registered in the PROSPERO register. PRISMA guidelines were followed in the reporting of this study.

**Eligibility Criteria:** We considered for inclusion randomized controlled trials (RCTs), observational studies, case reports, and case series, with tinnitus as primary outcome in children (0–18 years old) with acute or chronic subjective tinnitus. We excluded studies in which both children and adults participated but outcomes were not specifically reported for children, as well as animal studies, studies with a non-original study design and studies about children with pulsatile or objective tinnitus.

**Data Selection:** Two reviewers independently assessed studies for eligibility and quality, collected and extracted data. Statistical analyses were performed in case of homogeneous outcomes.

**Results:** The search yielded a total of 4,447 studies. Of these, 147 eligible studies were selected. One case report and five observational studies met the eligibility criteria. Three studies applied counseling and (simplified-)TRT and reported improvement in tinnitus outcome in 68 out of 82 children after 3–6 months of treatment. Two studies used pharmacological treatments and reported improvement in 74 out of 86 patients after 10 days to 3 months of treatment. One study reported the outcome of biofeedback therapy, describing an improvement in tinnitus loudness and annoyance after 2 months of treatment.

**Conclusion:** Due to the high risk of bias of the included studies, we cannot determine the effectiveness of the treatment of subjective tinnitus in children. Also, owing to brief follow-up periods, it is not possible to draw conclusions regarding long-term effects. Randomized controlled trials with longer follow-up periods are necessary to provide substantial evidence of the effects of therapies for children affected by tinnitus. https://www.crd.york.ac.uk/prospero/

**Systematic Review Registration:**https://www.crd.york.ac.uk/prospero/, identifier [CRD42020178134].

## Introduction

Tinnitus is the perception of a sound in the absence of an external source (1). It is often described as a ringing, rustling or buzzing sound. Tinnitus has the potential to become severe, negatively affecting quality of life and impairing normal daily activity in both adults and children (2). It can be classified as being objective or subjective. Objective tinnitus refers to a sound that the examiner can also hear during testing. Subjective tinnitus is the perception of a sound experienced by the patient for which no source can be found.

Despite the large number of studies about the prevalence, diagnosis and treatment of subjective tinnitus in adults, not much is known about tinnitus in children. Prevalence estimates range from 4.7 to 46% in children out of the general population and from 23.5 to 62.2% in children with hearing loss (3), with numbers depending on the definitions and characteristics of the studied cohort (4). Several risk factors have been found for pediatric tinnitus, including hearing loss, noise exposure, age and sex (with a greater prevalence found among older children and girls) (5). Stress, anxiety, and hyperacusis are also found to be associated with tinnitus (6, 7). Due to its effect on sleep, concentration and attention, hearing and emotional health, and tinnitus has the potential to negatively impact the lives of affected children (8).

For adults, several treatment modalities have been designed and are classically divided into pharmacological, sound and psychological therapies. Of the latter, cognitive behavioral therapy (CBT) has proven to be effective in improving the quality of life of those affected (9). It focuses on “confronting, disputing, and restructuring maladaptive thought patterns in order to develop more adaptive patterns of thought, leading to more adaptive emotional and behavioral responses” (10). Another psychological therapy such as Tinnitus Retraining Therapy (TRT) is mainly aimed at reclassifying tinnitus into a category of neutral percepts and at reducing the intensity of the tinnitus signal, through counseling and sound therapy, respectively (11). It is suggested that the treatment of tinnitus in children requires a similar approach with a focus on psychological aspects, reducing distress and awareness (12). Despite the fact that both the individual and the societal burden of tinnitus in children has drawn little attention in literature, therapeutic studies have been initiated for this group. To date, no clinical guidelines exist for the treatment of children with tinnitus. Therefore, our aim is to systematically review the treatment of subjective tinnitus in children and to assess the outcomes.

## Methods

### Protocol and Registration

We followed the PRISMA guidelines (Preferred Reporting Items for Systematic Reviews and Meta-Analysis) for this systematic review (13). The protocol for this systematic review was registered in the PROSPERO register (registration number CRD42020178134).

### Search Strategy

A systematic search was performed in MEDLINE and EMBASE. The most recent search was performed on 22 July 2021, using the terms “child” and “tinnitus” as well as synonyms. The search syntaxes are provided in the appendix. There were no restrictions concerning publication year, language or publication status of studies. In addition to electronic database searches, references and citations of the included studies were hand searched using Scopus databases to further identify any possible relevant studies.

### Study Selection

Following removal of duplicates the reviewers (MD, MK) independently performed title-abstract screening and full-text screening, using Rayyan as screening tool (14). If a full-text was not available, we contacted authors using ResearchGate and e-mail when possible. Studies published in languages other than Dutch and English were translated by a native speaker of that language. We considered for inclusion randomized controlled trials (RCTs), observational studies, case reports and case series, with tinnitus as primary outcome. We included studies providing outcomes for children with acute or chronic subjective tinnitus and describing pharmacological treatments as well as therapies aimed at reducing tinnitus burden or developing coping strategies aimed at managing tinnitus symptoms. We excluded studies in which both children and adults participated but outcomes were not specifically reported for children, as well as animal studies, studies with a non-original study design and studies about children with pulsatile or objective tinnitus, or tinnitus exclusively due to (sudden) sensorineural hearing loss. Conflicts were resolved through discussion with two additional reviewers (AS, IS).

### Data Collection and Analysis

#### Quality Assessment of the Studies

Each selected study was independently assessed for risk of bias by the reviewers (MD, MK), taking the following categories into consideration, as guided by the Cochrane Handbook for Systematic Reviews of Interventions (15): risk of bias due to confounding, selection of participants, classification of interventions, deviations from intended interventions, missing data and measurement of outcomes. Included cohort studies were assessed using the Risk of Bias in Non-Randomized Studies of Interventions (ROBINS-I) tool (16). This involved assessing each category and making a risk of bias judgment per category using the following grading system: “low,” “moderate,” “serious,” or “critical.” Following this, a final judgment regarding the quality of the study was made, using the same system. For a detailed explanation on how and why judgments of the risk of bias categories were made (see Supplementary Material). For clarification of the criteria that were used, see Tables C and D of the ROBINS-I tool. Disagreements were resolved by discussion. If no consensus was reached, we contacted the third author (AS).

#### Data Extraction and Synthesis

Original data from the selected studies were extracted by the reviewers (MD, MK). Information extracted included: author, year of publication, country of recruitment, study design, setting and study population, inclusion and exclusion criteria, total number and number of male and female participants, conflicts of interest, funding, intervention type and, if applicable, the dosage and method of administration, duration of treatment, adherence to treatment, co-interventions, follow-up time, definition of outcomes and statistical tests. Data extracted included: age (mean or range), gender, methods used to diagnose tinnitus and to grade its severity, duration and severity, the presence of risk factors and how they were measured, pre- and post-intervention scores, criteria used to define the outcome, loss to follow-up and attrition. Lastly, outcome data extracted included: number of participants for each treatment category before and after treatment and results of statistical group comparisons. Whenever necessary and if possible, these data were extracted from tables and figures. After extraction, data from the included studies were analyzed by the reviewers for disagreements, who discussed the studies in question to reach a consensus.

#### Outcome Measures

We analyzed studies reporting outcomes regarding perceptual measurements such as loudness and pitch, measured using numerical scales such as the visual analogue scale (VAS), or performance-based procedures such as tinnitus loudness or pitch matching (TLM and TPM, respectively). In addition, we considered studies that used self-formulated questions to diagnose tinnitus and to grade its severity. Furthermore, we analyzed studies reporting outcomes regarding impairment of daily activities and impact on quality of life, measured on a numerical scale such as the VAS or by a multi-item questionnaire. We considered the following multi-item questionnaires: Tinnitus Handicap Inventory [THI (17)], Tinnitus Handicap Questionnaire (18), Tinnitus Questionnaire (19), Tinnitus Reaction Questionnaire (20), Tinnitus Severity Scale (21) and the Tinnitus Functional Index (22).

#### Statistics

We extracted statistical data such as interquartile, mean or median values, as well as confidence intervals and *p*-values, whenever provided. If provided with sufficiently homogeneous outcomes, we intended to perform a meta-analysis.

## Results

### Results of the Search and Study Selection

**Figure 1:**
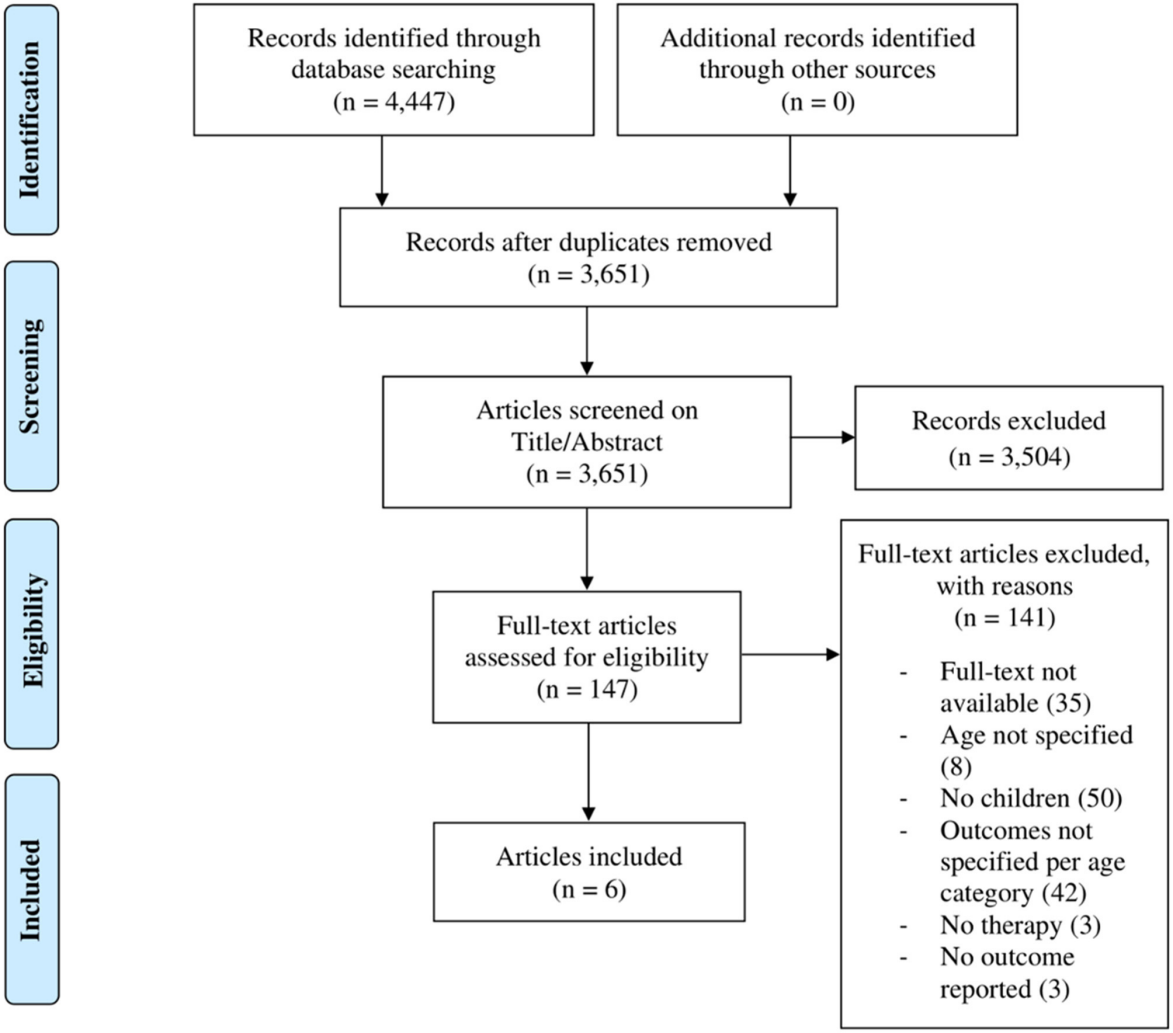
Study selection flow chart.

The electronic search yielded a total of 4,447 studies. After removal of 796 duplicates, 3,651 studies were screened on title-abstract, which resulted in 147 studies eligible for full-text screening. For 35 of the studies, the full-text was not available despite attempts to contact the authors. Full-text screening of the remainder resulted in 141 exclusions, for the following reasons: of 35 studies no full text was provided, 50 studies did not include children, 42 studies did not specify the outcomes for adults and children separately, three studies that were not therapeutic, three reported no outcomes and another eight studies did not specify the age of the study population. Of these, we contacted the authors, which yielded no further inclusions. Six studies were selected for analysis and data extraction (23–28). Four studies (23, 24, 26, 27) applied counseling of which three (23, 24, 27) additionally applied (simplified-)TRT (s-TRT). Two studies (26, 28) used pharmacological treatments and one reported on the outcome of biofeedback therapy, applied on one patient (25). One study was published in Polish (26) and another in German (28), and were translated to Dutch. No further eligible records were identified by hand searching reference lists of the included studies using Scopus databases for additional citations (Figure 1).

### Quality of the Included Studies

**Figure 2:**
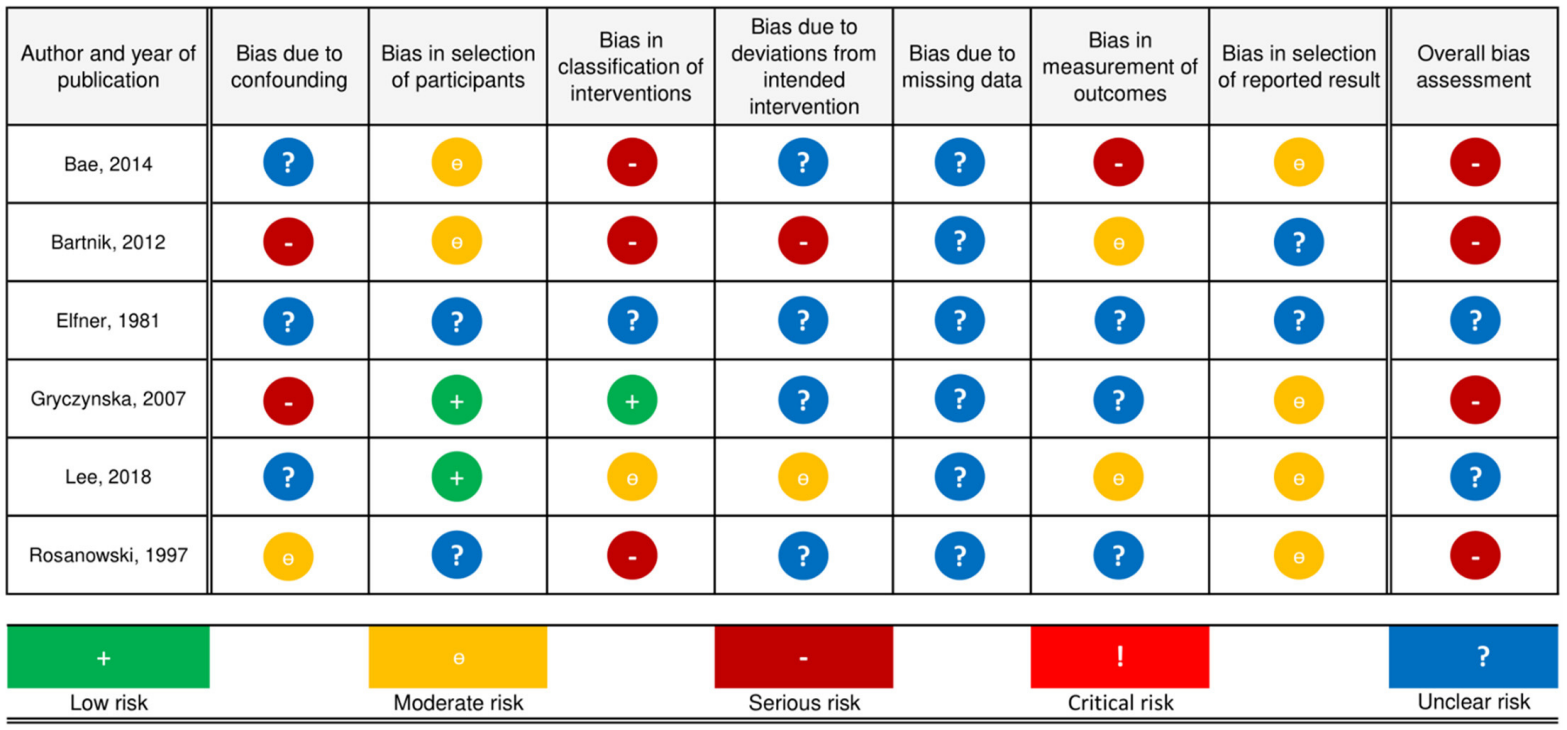
Risk of bias judgments.

Of the included studies, three were retrospective cohort studies (23, 24, 28), two were prospective cohort studies (26, 27) and one was a case report (25). A detailed discussion of risk of bias judgments is provided in the tables Risk of Bias Judgments, which can be found in the Supplementary Material. Overall, the risk of bias was serious in all observational studies (23, 24, 26–28), and the level of evidence is therefore IIa—(29). Because the study by Elfner et al. (25) is a case report, the ROBINS-I tool (16) was deemed unsuitable to make an accurate judgment regarding the risk of bias (Figure 2).

#### Confounding

Two studies (24, 26) had a serious risk of bias, one study (28) was at moderate risk of bias and it was unclear for three studies (23, 25, 27). None of the studies reported on the use of hearing aids in case of hearing loss or medications before the intervention was applied. Two studies (24, 26) reported that hyperacusis and anxiety were present, but outcomes were not reported separately for these patients, or adjusted for by design or statistical measures if possible. Rosanowski et al. (28) excluded participants with hearing loss, anxiety or mental symptoms, but did not screen for hyperacusis. Two studies did not mention hyperacusis or anxiety to be present among participants and did not exclude or treat separately those with hearing loss (23, 27).

#### Selection of Participants

Two studies (23, 24) had a moderate risk of bias due to their retrospective nature. Two studies (26, 27) had a low risk of bias, and for two studies it was unclear (25, 28).

#### Classification of Interventions

Three studies (23, 24, 28) were at serious risk, one study (27) at moderate, one study (26) at low risk and it was unclear for another study. Classification of intervention status was probably affected by tinnitus severity in three studies. Lee et al. (27) applied sound therapy but did not report which sound and to which degree it was applied. 26. Gryczyńska et al. (26) clearly defined treatment arms and treatment allocation.

#### Deviations From Intended Intervention

One study (24) was at serious risk of bias due to deviations from intended intervention(s), one study (27) at moderate risk of bias and in four studies (23, 25, 26, 28) the risk of bias was unclear. Bartnik et al. (24) performed myringotomy with tube insertion on two participants with either conductive or mixed hearing loss who also received the intended treatment, but did not report separate outcomes. In addition, less than half of the patients strictly observed the instructions relating to the treatment. Lee et al. (27) reported that patients were allowed to choose part of their therapy and use it to each one's individual need. For Rosanowski et al. (28), it was unclear whether follow-up time started before or directly after intervention, as follow-up time was not conceived beforehand and varied from 12 to 44 months.

#### Missing Data

In three studies (23, 26, 27) there was a substantial loss to follow-up, for which no reasons were reported. Bartnik et al. (24) reported no loss to follow-up but assessed the outcome of two participants as undefined result, without providing further elaboration. Rosanowski et al. (28) did not report information on missing data.

#### Measurement of Outcomes

One study (23) was at serious risk of bias, two studies (24, 27) at moderate risk and three studies (25, 26, 28) at unclear risk. Bae et al. (23) asked parents of children younger than 12 to answer the question by which the outcome was assessed. In two studies (24, 27) the outcome assessors were aware of the intervention received by the participants. In three studies (25, 26, 28) it was not reported how outcomes were assessed.

#### Selection of Reported Result

Four studies (23, 26–28) were at moderate risk and the risk of bias was unclear in two studies (24, 25). None of the included studies had a preregistered protocol available for inspection. In Bartnik et al. (24), the outcome was not provided for each child. In their results, Bae et al. (23) made no mention of how many children reported that their tinnitus had become worse after treatment.

#### Other Potential Sources of Bias

Two studies reported no conflicts of interest or funding (23, 26). Bae et al. (23) reported that counseling sessions were conducted by the author and it is unclear whether this possibly influenced the reporting of results.

### Data Extraction and Study Characteristics

#### Trial Design and Study Sample

Two studies were conducted in tinnitus clinics in South Korea (23) and Poland (24), one study in a pain and stress management center in the U.S. (25), one at a department of audiology in Germany (28) and two studies at an otorhinolaryngology department or outpatient clinic, one in Poland (26) and one in Japan (27). All studies were single-center and the total sample size was 479, with a range of 1–80 patients per study and an age range of 5–18 years of age (Table 1). All studies performed hearing assessments before treatment, using pure tone audiometry (PTA) (23–28) and tympanometry (23, 24, 26–28). A considerable methodological heterogeneity was found among the included studies due to different study designs, inclusion criteria, follow-up periods and the use of different tinnitus questionnaires. None of the studies performed statistical analyses of outcomes, therefore no confidence intervals or *p*-values were provided, and no meta-analysis could be performed.

**Table 1:** Study characteristics.

**Reference and country**	**Intervention**	**Study design and setting**	**Population**	**Exclusion criteria**	**Sample (no. of males)**	**Age (yrs)**	**Outcome**
Bae et al. (23) and South Korea	Counseling vs. s-TRT ± HA or SG	RCS, tinnitus patient clinic	I: mild tinnitus (*n* = 58) II: severe tinnitus (*n* = 15) III: tinnitus in quiet surroundings (*n* = 4) IV: tinnitus + HL (*n* = 3)	No tinnitus, SOM +, age > 18	80 (30)	5–18	Single question about tinnitus disturbance and frequency
Bartnik et al. (24) and Poland	TRT ± HA or SG	RCS, tinnitus patient clinic	I: tinnitus (*n* = 14) II: tinnitus + HL (*n* = 32) III: tinnitus + hyperacusis (*n* = 12), HL (*n* = 1)	Abnormal tympanometry test, inability to complete 6 months of treatment, hyperacusis as sole complaint, VAS < 5 on 3 parameters, age > 18	59 (28)	7–17	VAS on impact on daily activities, awareness, annoyance, and intensity and distress (0 = least; 10 = most)
Elfner et al. (25) and U.S.	BFT	CR, tinnitus patient clinic	HL in the contralateral ear (*n* = 1)	NA	1 (1)	16	Clinician interview about tinnitus loudness and annoyance
Gryczyńska et al. (26) and Poland	Betahistine po	PCS, outpatient clinic	I: tinnitus + HL (*n* = 23) II: tinnitus (*n* = 32), tinnitus + hyperacusis (*n* = 8)	Conductive hearing loss due to cerumen or SOM +, age > 18	67 (31)	6–18	Tinnitus loudness, reported by patients on a 4-point scale
Lee and et al. (27), and Japan	TRT + radio music	PCS, hospital	I: tinnitus (*n* = 13) II: tinnitus + HL (*n* = 228)	Tinnitus duration > 3 months, THI score of <17, age >18	241 (120)	15	THI for tinnitus annoyance
Rosanowski et al. (28) and Germany	Counseling vs. xylocaine iv	RCS, audiology department	I: tinnitus + HL (*n* = 24) II: tinnitus + HL (*n* = 7)	Tinnitus duration of >6 months, abnormal hearing, psychiatric symptoms, age > 18	31 (12)	6–17	Question about laterality, persistence, temporality, and quality and wellbeing

*s-TRT, simplified tinnitus retraining therapy; HA, hearing aids; SG, sound generators; RCS, retrospective cohort study; Roman numbers (I–IV), treatment arms; yrs, years; HL, hearing loss; SOM, serous otitis media;*

*TRT, tinnitus retraining therapy; VAS, visual analogue scale; BFT, biofeedback therapy; CR, case report; NA, not applicable; po, per os; PCS, prospective cohort study; THI, tinnitus handicap inventory; iv, intravenous*.

#### Interventions

##### Tinnitus Retraining Therapy

Three studies (23, 24, 27) used TRT as main therapy. Bae et al. (23) used a simplified form of TRT (s-TRT), which consisted of a single counseling session of 30 min and sound therapy for 3 months. They divided patients in four groups who received either counseling alone in case of mild tinnitus, or counseling followed by sound therapy for severe tinnitus. Those with severe tinnitus and hearing loss also received hearing aids. Those who were severely bothered in quiet surroundings received sound generators. In Bartnik et al. (24) all patients received TRT during 6 months, which consisted of a single directive counseling (i.e., counselor-centered) session and sound therapy with hearing aids, or bedside sound generators or sound generators behind the ears. The follow-up counseling was performed per the individual needs of the patient. Lee et al. (27) used TRT with a single counseling session followed by sound therapy with radio music for 6 months.

##### Biofeedback Training

Elfner et al. (25) described a treatment consisting of weekly electromyography (EMG) and biofeedback training, with concurrent relaxation therapy for 2 months.

##### Betahistine

Gryczyńska et al. (26) applied tinnitus treatment consisting of oral betahistine for a period of 3 months. Children who weighed more than 50 kg were given 48 mg betahistine daily. Those who weighed less received 32 mg per day.

##### Xylocaine

Rosanowski et al. (28) offered treatment consisting of either counseling for controllable tinnitus symptoms, or intravenous xylocaine for decompensated tinnitus (32), in which case the children received 2 mg xylocaine per kilogram body weight (500 ml 6%) intravenously for 10 days consecutively.

#### Outcome Measures

Three studies measured tinnitus severity before starting treatment (23, 24, 27), of which two used validated instruments, i.e., VAS (24) and THI (27). The third study (23) asked how often participants were bothered by tinnitus or if it disturbed their daily activities. Outcome was measured by a single question “Is your tinnitus better, same or worse after the treatment?” Three studies (25, 26, 28) did not mention how tinnitus was measured. Bartnik et al. (24) defined improvement as liberation of at least one daily activity previously impaired, or a decrease by a minimum of 20% in VAS of at least three of the following parameters: impact on daily activities, annoyance, intensity, awareness and level of distress. Lee et al. (27) performed a THI every 3 months. In Elfner et al. (25) outcome was measured by means of a self-report of the decrease in tinnitus loudness or the ability to ignore the sound. Gryczyńska et al. (26) assessed tinnitus outcome every 4 weeks. Improvement, remission, deterioration of or no change in tinnitus outcome was noted. Rosanowski et al. (28) evaluated outcome by asking the patients whether tinnitus had improved or if it had completely resolved. Follow-up time was not conceived beforehand. Final outcomes were measured at three (23, 26, 27), six (24), 12 (25), and 12–44 months (28) after therapy (Table 2).

**Table 2:** Outcomes.

**Reference**	**Mean/min. duration of tinnitus**	**Intervention**	**Criteria used to define effect**	**Intervention groups**	**Pre**	**Post**	**Time of measurement/FU**
Bae et al. (23)	Mean 15.4 (sd 24.5) mo	Counseling vs. s-TRT ± HA or SG	Question if tinnitus was better, worse or the same	I: Cs (*n* = 58) II: s-TRT (*n* = 15) III: s-TRT + SG (*n* = 4) IV: s-TRT + HA (*n* = 3)	I: mild tinnitus II–IV: severe tinnitus	I: 30 “better”, 2 “same”, 26 lfu II: 13 “better”, 2 lfu III: 4 “better” IV: 2 “better”, 1 lfu	After 3 mo treatment
Bartnik et al. (24)	50% <2 yrs	TRT ± HA or SG	Decrease of ≥20% in VAS on ≥3 parameters and liberation of ≥1 activity	I: TRT and SG (*n* = 14) II: TRT and HA + SG (*n* = 32) III: TRT and SG (*n* = 13)	VAS ≥ 5 on three parameters	I: 12 improvement, 1 no improvement, 1 lfu II: 28 improvement, 4 no improvement III 8 improvement; 4 no improvement, 1 lfu	Before, during and after 6 mo treatment
Elfner et al. (25)	NI	BFT	Improvement of tinnitus loudness or tolerance	BFT (*n* = 1)	NI	Improvement	At 2 mo treatment, FU 12 mo
Gryczyńska et al. (26)	NI	Betahistine po	Relief of tinnitus on a 4-point scale	I: betahistine (*n* = 23) II: betahistine (*n* = 32)	NI	I: 3 relief, 15 remission, 5 lfu II: 5 relief, 20 remission, 7 lfu	At 1, 2, and 3 mo treatment
Lee et al. (27)	≤ 3 mo	TRT + radio music	Decrease in THI	Radio music as sound therapy (*n* = 1)	THI 46	THI 36	At 1, 3, and 6 mo treatment
Rosanowski et al. (28)	≤ 6 mo	Counseling vs. xylocaine iv	Improvement in laterality, persistence, temporality, quality and wellbeing	I: Cs (*n* = 24) II: xylocaine (*n* = 7)	I: controllable tinnitus II: uncontrollable tinnitus	I: 24 improvement II: 4 remission, 3 improvement	After 10 days treatment, 12–44 mo FU

*min., minimum; sd, standard deviation; Roman numbers (I–IV), treatment arms; FU, follow-up; mo, month(s); Cs, counseling; s-TRT, simplified tinnitus retraining therapy; yrs, years; SG, sound generator; HA, hearing aid;*

*VAS, visual analogue scale; +, improvement; NI, no information; BFT, biofeedback training; THI, tinnitus handicap inventory*.

#### Effects of Intervention

##### Tinnitus Retraining Therapy

In the study of Bae et al. (23), 58 patients received counseling of whom 30 reported improvement, two reported no changes and 30 were lost to follow-up. Of the group that received s-TRT, 13 out of 15 patients answered “better” to the question “Is your tinnitus better, same or worse after the treatment?” Two patients were lost to follow-up. Of seven patients who received s-TRT with either sound generators or hearing aids, all but one who was lost to follow-up, reported improvement. Bartnik et al. (24) reported improvement in 12 of 14 patients who received TRT with bed-side sound generators. One patient reported no changes and for another the outcome was denoted as undefined. Of 32 patients who were treated with TRT, hearing aids or bed-side sound generators, 28 reported improvement and four reported no changes. Of the 13 patients treated with TRT and bed-side sound generators or sound generators behind ears, eight reported improvement and four reported no changes. One outcome was denoted by the authors as undefined. Lee et al. (27) reported the outcome for one child, who received TRT with radio music. This patient's THI score was 46 points before the intervention, 42 by 3 months and 36 at final follow-up at 6 months.

##### Biofeedback Training

Elfner et al. (25) reported on the use of biofeedback training in one patient. It was reported that the patient experienced less frustration and insomnia, however the “ringing in the ears” remained unaltered. After 2 months of treatment the patient reported no complaints regarding tinnitus loudness. Although still present, the patient was able to ignore the sound. A year after completion of the therapy, the tinnitus was still present but no longer experienced as bothersome.

##### Betahistine

Gryczyńska et al. (26) showed that 15 of 18 hearing-impaired children reported noise reduction and that three reported remission of the tinnitus. In 20 of 25 children without hearing loss improvement was noted and five reported remission.

##### Xylocaine

Rosanowski et al. (28) reported that all 24 patients who received counseling reported improvement of tinnitus symptoms. Of those who received xylocaine iv, four out of seven patients reported complete remission of tinnitus symptoms, three reported improvement of their tinnitus. One of these patients required additional psychotherapy due to concurrent mental disease.

## Discussion

### Summary of Main Findings

We reviewed the current evidence relating to the outcome of different therapies for subjective tinnitus in children. Three studies (23, 24, 27) assessed outcomes of TRT with or without hearing aids or sound generators, and reported an improvement of tinnitus outcome in 68 of 83 children after 3 (23) and 6 (24, 27) months of treatment. Biofeedback therapy showed improvement of tinnitus loudness and annoyance after 3 months and at 1 year follow-up in one patient (25). Another study (26) reported improvement of tinnitus outcome in 43 of 55 patients after 3 months of treatment with oral betahistine, with nearly 20% of patients reporting complete remission. Lastly, 31 patients treated with either counseling or intravenous xylocaine all reported improvement, with four patients reporting complete remission after 12–44 months follow-up (28).

There are several mechanisms that could explain these favorable outcomes. Relatively high loss to follow-up numbers were observed, ranging from 0 to 36% with an overall average of 20%. This may have resulted in an overestimation of treatment effects. It has also been suggested that tinnitus in children is often self-limiting (12). In two studies (27, 28), a minimum duration of 3 and 6 months was considered an inclusion criterion. Therefore, a natural course and cessation of symptoms before and during the study period could have improved outcomes, as similar phenomena have been seen in adults (33). Furthermore, each of the included studies was conducted in a hospital or clinical setting, after which the results were analyzed retrospectively. Also, none were randomized, leading to considerable risks of bias in the included studies.

Confounding was seen in three studies (24, 26, 28). Hearing loss and mental health issues such as anxiety are considered risk factors for tinnitus, and may thus act as a confounder (5, 6, 34, 35). Treating hearing loss or anxiety could ameliorate tinnitus (6, 36, 37), possibly leading to outcomes not solely attributable to the treatments given for the tinnitus. Nonetheless, in two studies, children received intervention for hearing loss concurrently, through either hearing aids (23) or myringotomy and tube insertion (24). In spite of the potentially confounding effect of anxiety, only one study (28) described excluding children with psychiatric symptoms after a psychiatric evaluation. Performing a randomized and blinded study would be beneficial to study outcomes of tinnitus therapy, by diminishing possible confounding by indication (38). Despite their prospective design and applying pharmacological therapies, two studies (26, 27) did not randomize participants, nor were they or the clinicians blinded, and the studies were not placebo-controlled. Furthermore, two studies (23, 24) treated patients, both with different types of TRT, in which assignment was based on a pre-intervention tinnitus score. As patients with a more severe form received more intensive therapy, this assignment could have influenced the outcomes of the intervention given (39).

The main difficulty in assessing effects of treatment of tinnitus in children, is the current lack of methodological standards and outcome measures to assess the severity and impact of, or the distress caused by, tinnitus in children (40, 41). Attempts at developing an objective method to measure tinnitus have not yet yielded any success (42). For adults, the TFI was created in 2012 and validated to measure treatment outcomes, encompassing domains such as sleep, hearing, mood and concentration (22). To date, no such instruments have been developed for measuring tinnitus in children (8). Over the past 4 years, several projects have been initiated to achieve an international standard in measuring tinnitus distress in adults such as the COMiT'ID initiative (43). Its members have created recommendations for core outcomes for tinnitus therapy for adults. Thus far, these projects have not yet been established for children. Furthermore, until now, there was only one previously published systematic review on the treatment of tinnitus in children (30). They included studies that provided data about tinnitus prevalence or management, and excluded non-English papers. They reviewed three studies, two of which are also included in this review (23, 24). One study included in this review was not selected for the current study because its primary aim was to treat hearing loss (44). In this review by Lee et al. (30) the authors stated that, because of the high rate of success of treating tinnitus in children with counseling only (93.8% reported improvement), counseling and conservative management may be sufficiently beneficial in children with mild tinnitus. This supports the argument that pediatric tinnitus can be self-limiting in many cases, but the authors underlined the high loss to follow-up numbers in this group. Due to lack of a baseline value of tinnitus severity in three of our included studies (25, 26, 28), it cannot be concluded to which degree of severity the described therapies are best suited. Whilst mild tinnitus may be self-limiting and counseling might thus prove to be sufficient, the scoping review by Smith et al. (8) shows that severe tinnitus can seriously reduce children's quality of life, which supports the view that young patients with more severe tinnitus do need treatment (12). As of yet, no treatment modalities have been developed for children specifically, although one study (23) modified an existing therapy to improve its practical applicability in children through a simplified form of TRT, in which only a single counseling session is mandatory and the emphasis is laid on sound therapy. As seen with certain psychotherapies that have been specifically designed and proven successful for children with pain (45) or depressive disorders (31), adapting existing therapies intended for adult patients could be a next step in adequately treating children.

### Limitations

Several methodological considerations have to be taken into consideration when systematically reviewing literature. Since MeSH terms and title and abstract terms are concise and not many synonyms exist, the search strategy for tinnitus and children studies is not complex. The screening was conducted by two screeners. We decided to include all types of studies, and not to restrict this review to randomized controlled trials because we want to give a broad view of the studies and the quality of the studies concerning children, tinnitus and treatments. Hence, this is the first systematic review which includes all of the current literature on the treatment of subjective tinnitus in children, and we hope to have provided a basis for further research on the subject.

## Conclusion

Due to the high risk of bias of the included studies, we cannot determine the effectiveness of the treatment of subjective tinnitus in children. Also, owing to brief follow-up periods, it is not possible to draw conclusions regarding long-term effects. Randomized controlled trials with longer follow-up periods are necessary to provide substantial evidence of the effects of therapies for children affected by tinnitus.

## Data Availability Statement

The original contributions presented in the study are included in the article/Supplementary Material, further inquiries can be directed to the corresponding author(s).

## Author Contributions

MD, AS, and IS designed the study. MD and MK conducted the search, selected the eligible studies and screened them, on title-abstract as well as full-text. Original data from included studies were extracted and analyzed by MD and MK, under the supervision and with the assistance of AS and IS. This manuscript was written by MD and MK and was critically revised and finally approved by AS and IS. All authors contributed to the article and approved the submitted version.

## Conflict of Interest

The authors declare that the research was conducted in the absence of any commercial or financial relationships that could be construed as a potential conflict of interest.

## Publisher's Note

All claims expressed in this article are solely those of the authors and do not necessarily represent those of their affiliated organizations, or those of the publisher, the editors and the reviewers. Any product that may be evaluated in this article, or claim that may be made by its manufacturer, is not guaranteed or endorsed by the publisher.
