# Supplementary material for: Treatment of Tinnitus in Children—A Systematic Review
Source: Front Neurol. 2021 Sep 10;12:726803. doi: 10.3389/fneur.2021.726803 (PMC8460757; doi:10.3389/fneur.2021.726803)
Supplement: Supplementary file 1 [file Table_1.pdf]

Supplementary Material

List the confounding domains relevant to all or most studies
Hearing loss, mental disorders or mental symptoms, hyperacusis.

List co-interventions that could be different between intervention groups and that could impact on outcomes
Medications, hearing aids, counseling pre-treatment or as part of the treatment, another intervention aimed at reducing a confounding domain.

Explanation of how risk of bias judgments were reached

Bae et al.

**Bias due to**	**Author's judgment**	**Support for judgment**
Confounding	Unclear	Possible causes of (otic) tinnitus were not listed, thus it is not clear whether confounding was present.
Selection of participants	Moderate	Considering the retrospective nature of the study, there is always a certain risk of bias to take into account.
Classification of interventions	Serious	Severity of tinnitus is one of the cornerstones of classification of intervention status. This means that there is a probability of recall bias, and also knowledge of the outcome of the intervention may have affected intervention status. Also, children are assigned to a certain intervention according to the severity of their tinnitus and/or hearing loss. Classification of intervention status was probably affected by knowledge of the outcome as children with more severe tinnitus received a more intense therapy.
Deviations from intended interventions	Unclear	No information was reported on whether there was deviation from the intended intervention.
Missing data	Unclear	High number of patients lost to follow-up, however no elaboration.
Measurement of outcomes	Serious	The outcome measure was subjective (i.e. vulnerable to influence by knowledge of the intervention received by study participants); and the outcome was assessed by assessors aware of the intervention received by study participants. Also, children younger than 12 years did not answer questions assessing outcome themselves.
Selection of the reported result	Moderate	There is no preregistered protocol available for inspection. In addition, there is no mention of one of the possible outcomes after treatment ('worse') in the results.
Other bias	No conflicts of interest or funding to declare, first author also partly performed interventions	
Overall	Serious	

Bartnik et al.

**Bias due to**	**Author's judgment**	**Support for judgment**
Confounding	Serious	No randomization. No psychiatric evaluation was performed to measure mental health issues. Children with and without hyperacusis and hearing loss are not mixed and receive different treatments, of which the outcomes are then compared.
Selection of participants	Moderate	Considering the retrospective nature of the study, there is always a certain risk of bias to take into account.
Classification of interventions	Serious	Classification of intervention status is based on tinnitus severity. Knowledge of the outcome of the intervention may have affected intervention status. Also, information about interventions was collected retrospectively.
Deviations from intended interventions	Serious	Some children with conductive hearing loss received myringotomy and tube insertion as co-intervention, others did not. It is also stated that less than half of the patients (47,5%) strictly observed the orders.
Missing data	Unclear	There are two children who demonstrated an undefined result, which is not further elaborated on.
Measurement of outcomes	Moderate	The outcome measure was subjective (i.e. vulnerable to influence by knowledge of the intervention received by study participants); and the outcome was assessed by assessors aware of the intervention received by study participants.
Selection of the reported result	Unclear	It states that in cat. I 13 of 14 children received bed-side sound generators. It reports 'significant improvement' in 12 out of 14 children, 'no improvement' in 1 child and 'undefined result' in another. However, it is unclear which child reported which outcome. Also, it is not reported what the outcomes were for the children who received co-interventions.
Other bias	Conflicts of interest and funding were not reported	
Overall	Serious	

Elfner et al.

**Bias due to**	**Author's judgment**	**Support for judgment**
Confounding	Unclear	NA
Selection of participants	Unclear	
Classification of interventions	Unclear	
Deviations from intended interventions	Unclear	
Missing data	Unclear	
Measurement of outcomes	Unclear	
Selection of the reported result	Unclear	
Other bias	Conflicts of interest and funding were not reported	
Overall	Unclear	NA

Gryczynska et al.

**Bias due to**	**Author's judgment**	**Support for judgment**
Confounding	Serious	No mention of confounding domains in the article: children with and without hearing loss are treated separately, however they excluded only children with conductive hearing loss. Also, hyperacusis and anxiety as risk factors are present but not measured and/or controlled for by randomization.
Selection of participants	Low	Selection of participants was not based on patient characteristics observed after start of the intervention.
Classification of interventions	Low	Groups and assignments to intervention were clearly defined.
Deviations from intended interventions	Unclear	There were no deviations from intended deviations reported.
Missing data	Unclear	Outcome data was not available for all patients, and there is no report of this loss to follow-up or its cause(s).
Measurement of outcomes	Unclear	It was not reported how outcome was assessed.
Selection of the reported result	Moderate	There is no preregistered protocol available for inspection.
Other bias	No conflicts of interest or funding to declare	
Overall	Serious	

Lee et al.

**Bias due to**	**Author's judgment**	**Support for judgment**
Confounding	Unclear	The possible causes of tinnitus were not listed, and there is no mention of anxiety as possible confounder, or mental evaluation in the article. No randomization.
Selection of participants	Low	All participants who would have been eligible for the target trial were included in the study and for each participant, start of follow up and start of intervention coincide.
Classification of interventions	Moderate	Patients chose the kind of sound they wished to hear during the retraining therapy and listened to it as much as they liked. They do not provide the reader with an estimation to which degree the therapy was applied.
Deviations from intended interventions	Moderate	Counseling was performed preintervention. It is unclear to which degree participants adhered to treatment.
Missing data	Unclear	Intended follow-up time was 18 months, however only 4 out of 13 were available after 12 months. No reasons for loss to follow-up were reported.
Measurement of outcomes	Moderate	The outcome measure was subjective (i.e. vulnerable to influence by knowledge of the intervention received by study participants); and the outcome was assessed by assessors aware of the intervention received by study participants.
Selection of the reported result	Moderate	There is no preregistered protocol available for inspection.
Other bias	Conflicts of interest and funding were not reported	
Overall	Unclear	

Rosanowski et al.

**Bias due to**	**Author's judgment**	**Support for judgment**
Confounding	Moderate	Confounding expected, all known confounding domains except hyperacusis mentioned and appropriately measured and controlled for. Screening for any mental disorders or psycho-symptomatology was performed, as well as a audiometry which revealed no hearing loss in both groups. No randomization.
Selection of participants	Unclear	Not clear whether start of follow-up and start of intervention coincided for each participant.
Classification of interventions	Serious	Children with mild (compensated) tinnitus received counseling alone, whereas children with severe (decompensated) tinnitus receive a more intense therapy.
Deviations from intended interventions	Unclear	History, physical and audiometric exams were performed for every patient. There were no deviations from interventions. Follow-up was not conceived beforehand and thus varied great between patients. It is not clear whether follow-up started before or after intervention.
Missing data	Unclear	No information is reported about missing data or the potential for data to be missing.
Measurement of outcomes	Unclear	No information about the method of outcome assessment.
Selection of the reported result	Moderate	There is no preregistered protocol available for inspection.
Other bias	Conflicts of interest and funding were not reported	
Overall	Serious	
